# Viral Persistence and Chronicity in Hepatitis C Virus Infection: Role of T-Cell Apoptosis, Senescence and Exhaustion

**DOI:** 10.3390/cells7100165

**Published:** 2018-10-12

**Authors:** Muttiah Barathan, Rosmawati Mohamed, Yean K. Yong, Meganathan Kannan, Jamuna Vadivelu, Alireza Saeidi, Marie Larsson, Esaki Muthu Shankar

**Affiliations:** 1 Department of Medical Microbiology, Faculty of Medicine, University of Malaya, LembahPantai, 50603 Kuala Lumpur, Malaysia; barathanmuttiah@gmail.com (M.B.); jamuna@ummc.edu.my (J.V.); alirezabcsaeidi@gmail.com (A.S.); 2 Department of Medicine, Faculty of Medicine, University of Malaya, 50603 LembahPantai, Kuala Lumpur, Malaysia; rosmohamed@um.edu.my; 3 Laboratory Center, Xiamen University Malaysia, 43900 Sepang, Malaysia; yong.yeankong@gmail.com; 4 Division of Blood and Vascular Biology, Department of Life Sciences, Central University of Tamil Nadu (CUTN), Thiruvarur 610005, India; meganathank@cutn.ac.in; 5 Division of Molecular Virology, Department of Clinical and Experimental Medicine, Linkoping University, 58 183 Linkoping, Sweden; marie.larsson@liu.se; 6 Division of Infection Biology and Medical Microbiology, Department of Life Sciences, Central University of Tamil Nadu (CUTN), Thiruvarur 610005, India

**Keywords:** apoptosis, viral persistence, hepatitis C virus, immunity, chronic infection

## Abstract

Hepatitis C virus (HCV) represents a challenging global health threat to ~200 million infected individuals. Clinical data suggest that only ~10–15% of acutely HCV-infected individuals will achieve spontaneous viral clearance despite exuberant virus-specific immune responses, which is largely attributed to difficulties in recognizing the pathognomonic symptoms during the initial stages of exposure to the virus. Given the paucity of a suitable small animal model, it is also equally challenging to study the early phases of viral establishment. Further, the host factors contributing to HCV chronicity in a vast majority of acutely HCV-infected individuals largely remain unexplored. The last few years have witnessed a surge in studies showing that HCV adopts myriad mechanisms to disconcert virus-specific immune responses in the host to establish persistence, which includes, but is not limited to viral escape mutations, viral growth at privileged sites, and antagonism. Here we discuss a few hitherto poorly explained mechanisms employed by HCV that are believed to lead to chronicity in infected individuals. A better understanding of these mechanisms would aid the design of improved therapeutic targets against viral establishment in susceptible individuals.

## 1. Introduction

Hepatitis C virus (HCV), first identified in 1989 as the causative agent of hepatitis C infection, is an RNA virus belonging to the genus *Hepacivirus* of the Flaviviridae family [1]. The genetic information of HCV is stored in RNA, which causes the virus to mutate rapidly, accounting for the expanding diversity of HCV with reports suggesting that there are as many as sevengenotypes and 90 subtypes of HCV [2]. Nearly1.75 million new cases were reported in 2015 by the World Health Organization (WHO), bringing the global total of people living with HCV to ~71 million [3]. HCV accounts for a majority of hepatocellular carcinoma (HCC) and liver transplantation cases worldwide. The infection has also been declared as a pandemic owing to its wider degree of geographic distribution and variability [4].

The very first drugs approved by the Food and Drug Administration (FDA) for HCV infection are PEGylated interferon alpha (peg-IFN) and ribavirin (RBV) that mainly target the virus rather than the host immune system [5]. The dosage and length of treatment are based on the HCV genotypes involved, for instance, HCV patients with genotypes 1, 4, 5 and 6 are treated for a period of over 48 weeks, whereas for genotypes 2 and 3 the period is only 12–24 weeks [6]. The recently designed oral regimens known as direct acting antivirals (DAA), contain a combination of several viral inhibitors that promise a shorter treatment duration with much higher cure rates, as well as fewer side effects. Nonetheless, there is a paucity of vaccines against all the variants of HCV, largely owing to the lack of a suitable small animal model or cell culture system [7].

HCV triggers inflammation of the liver, which ranges in severity from acute illness lasting for a few weeks to life-long chronic disease, termed as chronic hepatitis C, also known as persistent HCV infection [8]. However, due to the existence of several variants of HCV, the levels of potential persistence in the host and the susceptibility attributes to antiviral drugs may vary [9]. Epidemiological data suggest that ~15% of the infected individuals spontaneously clear the virus in the first sixmonths due to robust immune responses [10,11,12]. The biology of HCV chronicity and the potential mechanisms that harness viral persistence are poorly understood. Certain mechanisms have been postulated based onfindings from other chronic viral infections, such as human immunodeficiency virus (HIV) and hepatitis B virus (HBV). Here, we discuss certain hitherto poorly explored aspects of mechanisms employed by HCV in order to establish persistent infection, and also the potential strategies for preventing and reversing the immunological cues to favor viral control.

## 2. HCV Infection and Spontaneous Apoptosis

Apoptosis, or programmed cell death, represents an organized mechanism of cellular suicide key to removal of worn-out cells from the body [13]. Apoptosis is regarded as a host mechanism implicated in the pathogenesis of persistent viral infections and tumorigenesis [14]. On the contrary, growing evidence suggests that viruses tend to take advantage of the host cell machinery to induce apoptosis of tissues or immune cells as a way to delay virus-specific immune responses eventually leading to persistent infection [15]. Researchers have evidenced that in general, viruses use death receptor along with non-receptor signaling pathways by eliciting pro-apoptotic receptors or their ligands on the cell surface of infected individuals as a means to induce cell death, and eventually persistence [16].

Studies have shown that hepatocytes undergo apoptosis via up-regulation of death-inducing ligands CD95/Fas, TNF-related apoptosis-inducing ligand (TRAIL) and tumor necrosis factor alpha (TNF-α) on the cells during chronic HCV infection although the rates of apoptosis could vary between HCV genotypes [17,18]. In vitrostudies have shown that HCV structural and non-structural (core, NS4B and NS5B) proteins prompt cell death in liver cells via TNF-α by suppressing anti-apoptotic nuclear factor-κB (NF-κB) and activating stress c-Jun N-terminal kinase (JNK) pathways, leading to mitochondrial apoptosis [19,20]. This mechanism appears to be one reason for HCV-associated liver injury leading to liver cirrhosis and HCC during persistent HCV infection. Another study has postulated that certain HCV structural proteins could function as immunomodulators with up-regulated FasL on hepatocytes, causingactivated peripheral T-cells to undergo apoptosis via caspase 3 activation [21], eventually suppressing antiviral responses to assist viral persistence. It also appears that HCV not only infects hepatocytes but also B and T-cells, which accounts for the loss of optimal T-cell functions. Research also suggests that the augmented expression of Fas on peripheral T-cells of chronicallyinfected individuals relative to healthy controls, indicates the likely onset of T-cell apoptosis through the activation of caspase 8 and BH3 interacting-domain (Bid) death agonist precursors of death signaling in receptor-mediated pathways [22]. It is also becoming increasingly clear that the loss of HCV-specific CD8^+^ T-cells could delay the elimination of HCV-infected cells and virus neutralization [12]. Meanwhile, non-receptor-mediated apoptosis involving mitochondria, where livers of patients with chronic HCV infections show elevated reactive oxygen species (ROS) levels suggestive of caspase activation, apoptosis and peripheral T-cell deletion, has also been described [23]. Another study has shown that HCV infection could induce hepatocyte death via a mitochondrial cell death pathway, whereby activation of caspase 3, nuclear translocation of activated caspase 3, secretion of ROS, and release of cytochrome C from mitochondria were witnessed [24].

Recently we showed that several pro-apoptotic (TNF-α, FasL and TRAIL) and anti-apoptotic (BCL2L1, BCL2L2, and MCL-1) molecules were upregulated in chronic HCV infection, suggesting the presence of cell death in immune cells via the extrinsic and intrinsic pathways [25]. We also found excessively higher levels of TRAIL expression in our chronic hepatitis C (CHC) cohort, suggesting the onset of apoptosis. Establishment of apoptosis is further strengthened by the up-regulation of Bcl-2 family members in the peripheral blood mononuclear cells (PBMCs) of chronic HCV-infected patients. The Bcl-2-regulated pathway appears to be a vital trigger behind the induction of intrinsic and extrinsic apoptosis, which synergistically regulates apoptosis. We also hypothesized that the release of intracellular ROS in PBMC cultures derived from chronic HCV-infected patients, which likely initiates specific BH3-only proteins, inactivates BCL-2-like pro-survival molecules. We also found that BCL-2—an anti-apoptotic molecule—was down-regulated in chronic HCV infection [25]. We are anticipating that the stimulation of PBMCs of chronic HCV-infected patients with native HCV proteins could further underpin the association of virus-specific immune cells with apoptosis signaling and viral persistence.

## 3. Immunosenescence

During normal human ageing, the host immune system becomes poorly functional in terms of cellular responses towards foreign antigens, leading to poor naïve T-cell turnover and increased expansion of senescent T-cell phenotypes through a series of events called immunosenescence [26,27]. Studies have shown that during normal ageing, T-cells become increasingly vulnerable to immunosenescence resulting in a poor frequency of functional naïve T-cell repertoire, increased rates of differentiation of naïve T-cells to terminally-differentiated T-cells, telomere shortening, decline in CD4/CD8 ratios and increased numbers of memory T-cells lacking CD28 co-stimulatory molecule, collectively culminating in increased rates of susceptibility to infections, autoimmune disorders, chronic inflammatory diseases and cancers [28,29,30]. However, it has been shown that certain chronic viral infections, especially HIV and HBV have a clear role in necessitating immunological impairment via a process known as replicative senescence [31]. The mechanism occurs due to persistent exposure of T-cells to viral proteins resulting in chronic immune activation (CIA) [32]. Replicative senescent T-cells generally tend to display poor levels of expression of co-stimulatory molecules (CD28 and CD27) as well as T-cell survival molecules (CD127) [33]. Together, these cells also have an increased expression of CD57, a biomarker of replicative senescence [27] as well as an over-expression of chronic activation markers such as CD38 and HLA-DR [33]. Others have reported that the frequencies of CD28^+^CD57^+^ T-cells were higher among HIV-infected individuals relative to HIV seronegative individuals [34], which could be linked to increased resistance to apoptosis and poor rates of proliferation of CD8^+^ T-cells in HIV infection [35]. Subsequent work on HIV also points to the increased rates of recruitment of intermediately-differentiated CD4^+^ T-cell subsets (CD28^+^CD27^−^), suggesting the onset of immunosenescence [36]. The importance of CD127 expression on T-cells for survival and cytokine responses, especially during chronic HIV infection, has also been recommended [37]. Others have also postulated that HLA-DR expression on CD8^+^ T-cells is suggestive of excessive CIA [38,39]. Elevated levels of CD4^+^CD28^−^ T-cells co-expressing CD57 is suggestive of an expansion of senescent T-cells in chronic HBV infection [40]. Interestingly, a similar phenomenon has also been observed among chronic HCV-infected patients where higher proportions of effector senescent CD8^+^CD57^+^ T-cells were seen, which also appears to be markedly higher in liver cirrhosis [41]. Meanwhile, P16^INK4a^, a tumor suppressor protein, which is also regarded as an aging marker has been found to be elevated among individuals infected with and co-infected with HIV/HCV suggesting the likely role of immune aging during chronic HCV infection, which likely facilitates HCV persistence [42].

We have found that there is a paucity of knowledge on the potential role of immunosenescence, especially on the phenotypes of senescent T-cells in chronic HCV infection. Hence, we studied senescent T-cell phenotypes potentially associated with exhaustion in HCV-specific and non-specific T-cells, and also involving only individuals <53 years to avoid potential false-positive results [43]. In our study, HCV-specific CD4^+^ and CD8^+^ T-cells from chronic HCV-infected individuals showed a relatively increased expression of HLA-DR and CD38 as compared with T-cells derived from uninfected healthy controls. In addition, we found a clear up-regulation of CD57 on HCV-specific T-cells, which could likely be a physiological response to persistent antigenic exposure during chronic HCV infection and immune activation. The CD4^+^ and CD8^+^ T-cells from HCV-infected individuals showed a significant increase of late-differentiated T-cells, and reduced expression of co-stimulatory receptors (CD28 and CD27) as suggestive of immunosenescence [43]. The two co-stimulatory molecules, CD27 and CD28, are also associated with T-cellproliferation and are classical modulators of T-cell functions [44]. Our study also showed that expression of CD127 in T-cells of individuals with chronic HCV was dramatically reduced as compared with uninfected controls, suggesting that the function of T-cell subsets in activation, balance, differentiation, and survival could have been impaired in chronic HCV infection [45]. Further studies may be warranted to further elucidate the association of virus-specific T-cell senescence with functional responses in chronic HCV infection.

## 4. Immune Exhaustion

During viral infection, antigen presenting cells (APCs) such as dendritic cells will present virus derived peptide antigens to naïve CD4^+^ T-cells, which in turn will produce IL-2, IFN-γ and TNF-α following activation. This helps the short-lived cytotoxic T lymphocytes (CTLs) to differentiate into memory and effector CD8^+^ T-cells, culminating in the killing of viral-infected cells by secreting perforin and granzyme [46,47]. However, there is a completely contrasting phenomenon termed T-cell exhaustion, whereby the functions of both T-cell subsets in some viral infected patients will be impaired while causing a chronic inflammatory state as well as repeated stimulation of T-cells [48]. T-cell exhaustion was first described in the murine chronic lymphocytic choriomeningitis virus (LCMV) clone 13 model 49, where the phenomenon could eventually repress protective humoral as well as cell-mediated immune responses [48,49,50] towards viral infected cells by exhibiting sub-optimal proliferative abilities and cytokine production. Researchers have proposed that T-cell exhaustion frequently occurs in suppressive tumor microenvironments [51]. Another important hallmark of T-cell exhaustion of CD8^+^ and CD4^+^ T-cells is the up-regulation of inhibitory molecules/negative immune checkpoints that bind to their cognate ligands expressed on APCs during infection with adenovirus, HIV, HBV and HCV [52]. Exhausted T-cells express a group of co-inhibitory molecules (PD-1, TIM-3, CTLA-4, LAG-3, 2B4, BTLA and TRAIL) and reveal distinctive patterns of cytokines including anti-inflammatory, as well as pro-inflammatory cytokines (IL-2, TNF-α, I L-10 and TGF-β), besides transcriptional repressors, BLIMP-1 and FOXP3 [53]. A recent study has concisely shown that T-cell exhaustion is a part of the natural history of chronic HCV infection [54].

The seemingly very first study on the expression of exhaustion markers in HCV infection demonstrated that PD-1 expression on HCV-specific T-cells was relatively high during acute infection and a large part of the cells were shown to express PD-1 in chronic stages of HCV infection although this was irrespective of the clinical outcome of HCV disease [55,56]. In another study, researchers demonstrated that T-cells in the spleen of HCV-infected patients with cirrhosis expressed higher level of PD-1 and TIM-3, predominantly in the effector memory subpopulation [54]. It has also been shown that these markers were higher on T-cells derived from the spleen as compared with peripheral blood [54].Interestingly, markers of exhaustion were reduced in splenic CD8^+^ T-cells and APCs after the HCV-infected patients with cirrhosis underwent splenectomy, suggesting that splenic T-cells may have a role in controlling HCV [54,57]. Meanwhile, it has also been shown that high levels of PD-1 on peripheral CD8^+^ T-cells of chronically-infected HCV patients correlated with higher viral loads [58], suggesting that PD-1 may have a likely role in CD8^+^ T-cell dysfunction causing viral persistence. Another study on T-cell exhaustion in chronic HCV infection suggested that T-cells undergo rapid exhaustion with sequential loss of IL-2 production, proliferation and IFN-γ production [59,60], and increased expression of TIM-3, PD-1 and CTLA-4 [61] immediately following acute infection and persisting for a longer duration thereafter. When we compared the expression of co-inhibitory receptors and secretion of Th1/Th2/Th17 cytokines in T-cell cultures derived from chronic HCV patients and healthy controls, we found that HCV peptide activated CD4^+^ T-cells of chronic HCV patients expressed higher levels of PD-1 while CD8^+^ T-cells had expressed significant levels of TIM-3. CTLA-4 was seen to be expressed equally on both T-cell subsets [62]. In addition, secretion of IL-10 and TGF-β1 in T-cell cultures of chronic HCV patients were significantly increased, which are classically known to inhibit virus-specific T-cell expansion, cytokine production, and interfere with Th1/Th2 cell differentiation [63]. We proposed that accumulation of exhausted HCV-specific CD8^+^ and CD4^+^ T-cell subsets during chronic HCV infection results in considerable loss of protective functional immune responses likely contributing to HCV persistence [62].

## 5. Does Loss of MAIT Cells Contribute to HCV Persistence?

Specialized, innate-like T-cells with antimicrobial properties called mucosal-associated invariant T (MAIT) cells, represent an evolutionarily conserved αβ T-cell subset in humans, and are found in copious levels in the systemic circulation accounting for ~1–8% of the total T-cell pool of healthy adults [64]. MAIT cells can also be seen in lung and liver tissues (~50% of liver T-cells). MAIT cells express an invariable TCR-α chain (Vα19-Jα33 in mice and Vα7.2-Jα33 in human) together with a limited number of TCR-β chains (Vβ2 (TRBV20) and Vβ13 (TRBV6)) in humans and (Vβ6 and Vβ8) chains in mice [65]. MAIT cells share phenotypic resemblance with invariant natural killer T-cells (iNKT) and express high levels of IL-12Rβ2, IL-18Rα and CD161 together with the semi-invariant Vα7.2 segment [66]. The cells are readily activated by IL-12 and IL-18 along with some foreign antigens, usually microbe-derived vitamin B metabolites, which are presented by monomorphic MHC-I-like related protein (MR1) with the help of co-stimulatory molecules, CD80 or CD86, although MAIT cells are not part of cell-mediated immune responses [67,68]. Upon activation, MAIT cells tend to secrete a wide range of cytokines (IL-4, IL-5, IL-10, IFN-γ, TNF-α, IL-17 and IL-22) or release perforin as well as granzymes that could kill microbe-infected cells [69,70,71,72]. Several studies have proposed that MAIT cells can be associated with a number of conditions, including bacterial infections, inflammation-related diseases such as multiple sclerosis, psoriasis, allergy, autoimmune diseases and cancer [73,74]. Recent studies have demonstrated that although MAIT cells play a key role against viral infections, they do not directly recognize virally-infected cells.However, a cocktail of several innate cytokines (IL-18, IL-12, IL-15 and IFN-α) released from infected cells stimulate MAIT cells [75]. Researchers have shown that MAIT cells are severely diminished during chronic HCV infection, and represent exhausted and immune-activated phenotypes. In addition, MAIT cell populations reconstitute poorly in patients despite successful HCV clearance [76]. Another study has shown a decrease in the frequencies of CD161^++^Vα7.2^+^ T-cells in the peripheral blood of HIV/HCV co-infected patients [77]. Interestingly, others showed that although anti-viral therapy initiated in patients helps to increase the frequencies of MAIT cells in liver, it did not do so in the peripheral blood [78]. In one of our recent investigations, we found that MAIT cells in chronic HCV patients express high levels of CD57 besides PD-1, CD38 and HLA-DR. We postulated that peripheral MAIT cells undergoing exhaustion and senescence due to repeated activation of cells during chronic HCV infection could result in diminished innate defense attributes likely contributing to viral persistence and HCV disease progression [43].

## 6. Conclusions

In this review we have presented several investigations on factors causing HCV persistence in humans. Further, several recent investigations hint at the role of microRNA in HCV disease pathogenesis [79], for instance miR-146a-5p has been proposed to promote HCV infection through metabolic pathways associated with HCV-induced liver disease progression [80]. Meanwhile, miR-122 has been proposed to play a role in delaying virus-specific immune responses leading to progression of HCV malignancy [81]. Although several investigations have been conducted on apoptosis of activated immune cells through extrinsic and intrinsic apoptotic pathways, further studies may be paramount to improve understanding of the additional roles of co-inhibitory receptors in T-cell apoptosis other than in T-cell exhaustion. Furthermore, future studies should be aimed at addressing the role of the tissue-homing abilities of MAIT cells during viral infections. Studies should also emphasize how these mechanisms work in the context of phenotypic and functional immune cells during the course of acute and chronic infections. Lastly, the development of clinical immunotherapeutic agents such as dual blockade of co-inhibitory receptor pathways to overcome HCV-specific CD4^+^ and CD8^+^ T-cells along with MAIT cell exhaustion in chronic HCV infection, is an area that requires in-depth investigation.
